# An Analysis of Different Distance-Linkage Methods for Clustering Gene Expression Data and Observing Pleiotropy: Empirical Study

**DOI:** 10.2196/30890

**Published:** 2022-06-17

**Authors:** Joydhriti Choudhury, Faisal Bin Ashraf

**Affiliations:** 1 Brac University Dhaka Bangladesh

**Keywords:** gene clustering, gene expression, distance metric, linkage method, hierarchical clustering, pleiotropy

## Abstract

**Background:**

Large amounts of biological data have been generated over the last few decades, encouraging scientists to look for connections between genes that cause various diseases. Clustering illustrates such a relationship between numerous species and genes. Finding an appropriate distance-linkage metric to construct clusters from diverse biological data sets has thus become critical. Pleiotropy is also important for a gene’s expression to vary and create varied consequences in living things. Finding the pleiotropy of genes responsible for various diseases has become a major research challenge.

**Objective:**

Our goal was to establish the optimal distance-linkage strategy for creating reliable clusters from diverse data sets and identifying the common genes that cause various tumors to observe genes with pleiotropic effect.

**Methods:**

We considered 4 linking methods—single, complete, average, and ward—and 3 distance metrics—Euclidean, maximum, and Manhattan distance. For assessing the quality of different sets of clusters, we used a fitness function that combines silhouette width and within-cluster distance.

**Results:**

According to our findings, the maximum distance measure produces the highest-quality clusters. Moreover, for medium data set, the average linkage method, and for large data set, the ward linkage method works best. The outcome is not improved by using ensemble clustering. We also discovered genes that cause 3 different cancers and used gene enrichment to confirm our findings.

**Conclusions:**

Accuracy is crucial in clustering, and we investigated the accuracy of numerous clustering techniques in our research. Other studies may aid related works if the data set is similar to ours.

## Introduction

A substantial amount of genetic data began to accumulate in the hands of bioinformatics experts at the turn of the 21st century. The process was sped by advances in technology hardware and improved computer algorithms. Scientists began storing all of this genomic information in sequential data [1] and intensity matrix [2] formats. Different types of sequences, such as protein, DNA, and RNA sequences, are kept in sequential data format, and the intensity matrix preserves gene behavior under various conditions. To record and analyze gene behavior on sample individuals, these conditions can vary under varied light intensities.

Microarray [3] is a type of intensity matrix in which each row represents a single gene, and each column indicates that gene’s behavior in a given situation. A microarray data set’s sample structure is shown in Table 1. Four genes express themselves at 3 different times or circumstances. Depending on the normalization approach used, the values stored in a microarray data set can be both positive and negative.

**Table 1:** Sample microarray data.

Genes	Time 1	Time 2	Time 3
Gene 1	0.25	0.22	0.65
Gene 2	–0.75	1.25	–0.63
Gene 3	0.05	0.66	0.75
Gene 4	1.25	–0.52	0.15

Researchers have been extracting valuable biological information from microarray data. The construction of a phylogenetic tree is one of the most extensively used methodologies [4]. The evolutionary relationships between numerous species are shown by the phylogenetic tree. In the case of genes, it calculates gene similarity to create a gene tree that depicts how particular genes have evolved [5]. Although phylogenetic trees are based on sequence data because mutations occur in any species’ genome sequence, genome sequences are comparatively large and need a lot of computing power and memory. Gene expression represents phenotypes of a gene, and different genes exhibit variable levels of expression under the same conditions [6]. As a result, we can employ phenotype, which is a measurement of the genes’ reflection due to genotype differences. The expression level of genes calculates how near they are to one another using the microarray data set as an input, because the transcriptional activity of similar genes should be similar [7]. A tree is built by connecting all closely related genes one by one, with each leaf representing a single gene and branches separating one group of genes from another [8,9]. This hierarchical tree can aid in the creation of more precise groupings. It assists biologists in determining and comprehending the function of an unknown gene. As a result, developing appropriate metrics for clustering microarray data is a significant scientific challenge.

Different clustering approaches have been presented to extract information from the microarray data set [10]. Clustering algorithms divide unclassified data into distinct classified groups [11], with the most comparable data points grouped together. As a result, if an unknown element belongs to a recognized cluster, it becomes easier for the researcher to forecast its properties. Clustering is a technique used in bioinformatics to organize microarray data and predict properties of unknown genes based on which cluster they belong to [11]. Furthermore, bioinformatics workflow [12] and immune repertoire profiling [13] are classified using hierarchical clustering, a sort of clustering technique. It also has applications in the prediction of nonsmall cell lung cancer metastasis [14], the high-confidence identification of B cell clones [15], and the identification of cell type from a single cell transcriptome [16]. It is also used to create a phylogenetic tree using microarray data [15]. The hierarchical clustering methodology uses a distance algorithm to calculate the distance between distinct genes after inputting microarray data. The distance is then used to connect closely related genes in clusters using a linkage approach.

Various distance methods are employed depending on the data set’s characteristics. The way the 2 distance methods determine the difference between 2 distant data points is the fundamental distinction between them. Euclidean [17], Chebyshev [18], and other distance approaches are common. After applying the distance approach, the hierarchical clustering technique connects related genes using several types of linking methods to form a cluster. single linkage method [19], complete linkage method [20], average linkage method [20], and others are some of the most used linkage methods. Linkage methods connect genes in a bottom-up manner, eventually resulting in a hierarchical tree, often known as a phylogenetic tree. As computational ability and technology progress, it has become increasingly important to establish reliable clusters of related genes to understand unknown genes in sensitive domains such as health care and disease prediction.

Pleiotropy is another key phenomenon identified in the investigation of gene functions behind many diseases. Pleiotropy occurs when a single gene influences many phenotypic features [21]. There are numerous examples of multiple genes working together to cause a single disease [22-24]. Furthermore, it appears that a single gene is responsible for several disorders [25]. Even though we can identify diseases caused by the same gene, the gene’s impact on each disease is different. It may appear to be more active in some disorders than in others. As a result, we can visualize the impact of a gene on other diseases if we can detect commonalities in their expressions for different diseases and quantify the distance.

In this work, we used a variety of data sets to investigate different distance-linkage combinations for hierarchical clustering. These clusters have revealed which gene groupings are closely connected to one another. We also assessed the fitness of those groupings and attempted to determine which distance-linkage combination produced the greatest results. We validated our findings using 8 different data sets. Furthermore, we used the best measure to identify common genes responsible for various tumors. Gene enrichment scores about their influence on various diseases were used to corroborate our findings.

## Methods

This section goes over our proposed methodology. First, we provided the proposed workflow for determining the optimum clustering distance-linkage approach. Then we went over several distance metrics, linkage methods, and our selection procedure for comparing the performance of various combinations. Finally, the pleiotropic gene observation methodology is discussed.

### Identifying the Best Distance-Linkage Method

Our investigation begins with the import of a microarray data set into our procedure. This microarray data set is typically a 2D array, with rows representing different genes and columns representing their intensity at various time stamps. To minimize the dimensionality of the data set, we will use Principal Component Analysis. It is a sophisticated approach used by academics to remove irrelevant data from a data set while keeping its integrity.

**Figure 1:**
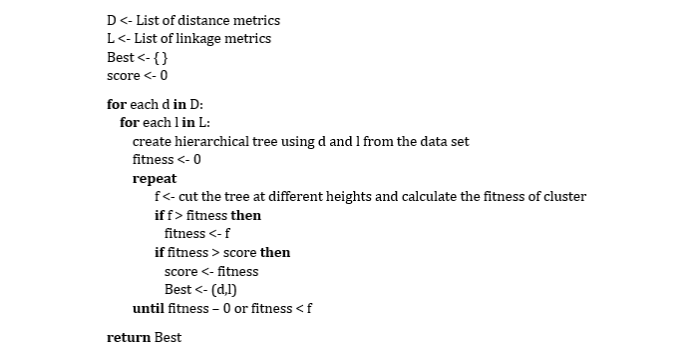
Proposed algorithm for finding the best distance-linkage combination. Input: Microarray data set. Output: Distance-linkage combination.

Then, in our data set, we run a distance metric. A distance measure, in general, calculates the similarity of 2 genes and determines how far apart they are. We employed the following 3 different distance metrics: Euclidean, Manhattan, and maximum. We chose a linkage method to connect related genes and generate a hierarchical tree after picking the distance metric. We used the following 4 linkage methods: single, complete, average, and ward linkage methods. We constructed a hierarchical tree using the distance-linkage method, where each leaf represents a gene, and the branches reflect the dissimilarity among them. The tree was then cut to various heights, resulting in several sets of genes for each cut point. Subsequently, we identified the appropriate cut point for that hierarchical tree by calculating how well those genes are clustered on different cut points. We used “Average Silhouette Width” and “Distance within Cluster” to calculate the fitness of the groups formed by different cut locations. The optimal fitness value is calculated using these fitness values. We determined the best combination of distance and linkage methods for a single data set by repeating this process with different combinations of distance and linkage methods. Figure 1 depicts the algorithm.

For a particular data set, D, optimal fitness value can be expressed by the following equations:




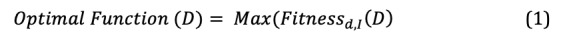




Where d 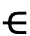 distance methods and, I 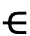 linkage methods.

#### Used Distance Methods

Euclidean distance uses Pythagorean formula to calculate the distance between 2 genes. For n dimensional space, we can write that formula as follows:




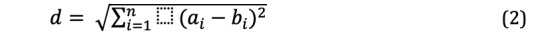




Unlike Euclidean distance, Manhattan distance takes the modulus value of the subtraction. For n-dimensional space, the equation of Manhattan Distance will be as follows:




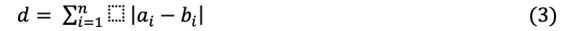




Maximum distance, on the other hand, calculates the subtraction value for each column before selecting the highest number. The formula for n-dimensional space is as follows:




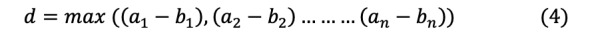




#### Used Linkage Methods

The single linkage approach connects 2 clusters by taking the shortest distance between them. The equation for the single linkage method to calculate the distance between any element and another element in another group is as follows:




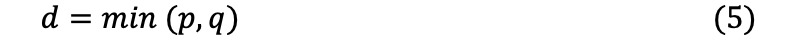




Where p is an element in cluster P and q is an element of cluster Q.

To compute the distance, the complete technique uses the farthest points in 2 clusters and connects the clusters with the shortest distance. The equation for the entire linking approach is as follows:




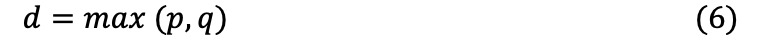




The average method determines the average value for each gene inside the cluster, then connects them one by one on each layer to form a hierarchical tree. Equation 7 is the average linkage method update formula.




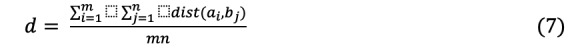




Where m is all the instances of cluster a, and n is all the instances of cluster b.

A centroid point is determined using Ward linkage (much like the centroid method). The squared distance value of each point in each cluster is then calculated using that centroid. It then sums all the squared distance values obtained by the 2 clusters together. It takes the smallest total value produced by a cluster pair and merges them on that level after repeating the same technique for every cluster on the same level. Equation 8 is the Ward linkage method update formula.




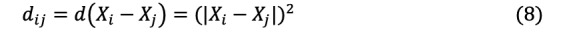




#### Metrics Used to Calculate Fitness

The fitness of the clusters we acquired after cutting the hierarchical tree at a specific height was calculated using the following 2 metrics: average silhouette width (ASW) and distance inside cluster. The following formula is used to compute silhouette width:




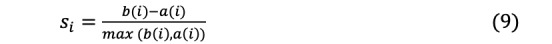




Where a(i) is the average distance from object i and all the other points of the cluster in which i belongs; b(i) is the distance of the closest point in other cluster; and s(i) is the silhouette value between 2 clusters.

ASW is the average of all the silhouette values. Generally, it varies from –1 to 1, and the value closer to 1 is considered better.

The distance within a cluster is used to determine how close the elements are. Each cluster’s centroid is chosen during this process. The distance between each object in the cluster and the centroid is then determined as an average. This calculation’s formula is as follows:




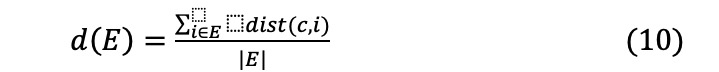




Where dist(c,i) is the distance between centroid c and element i in a cluster; E is the set of elements in the cluster; and |E| is the number of elements in the cluster.

From the characteristics, we can understand that ASW measures the quality of clusters. A greater ASW indicates good quality of clusters, that is, for a data set D, distance metric d and linkage method l,




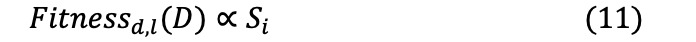




Where S_i_ is the ASW for cut point i.

However, distance within clusters measures how compact the data points are in the clusters. Therefore, better-quality clusters will have lower distance within clusters, that is,




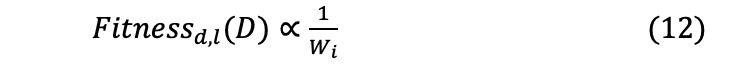




Where W_i_ is the distance within clusters for cut point i.

Thus, to compare the quality of clusters we acquired at different cut points i in the hierarchical tree, our fitness function combines these 2 criteria. When these 2 relationships are combined, our fitness function becomes as follows:




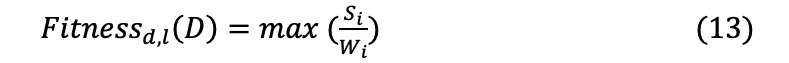




From this function, we can find out the optimal fitness for a specific combination of metrics in a certain data set.

### Cluster Ensemble

We will try ensemble clustering [26] to see if it works better once we have tried different clustering combinations. Three ensemble clustering techniques were employed, which are as follows: (1) similarity partitioning based on clusters; (2) hypergraph partitioning algorithm [27-29]; (3) meta-clustering algorithm.

#### Cluster-Based Similarity Partitioning

It starts by creating an n×n binary matrix in which the input is 1 if two objects belong to the same cluster and 0 otherwise. Every clustering approach is put through it. The final ensemble cluster is then generated using an entry-wise average of all clustering approaches.

#### Hypergraph Partitioning Algorithm

The data set is represented as a hypergraph by this algorithm. The hypergraph is then partitioned to determine the smallest number of edges. It produces the ensemble cluster based on the smallest number of edges.

#### Metaclustering Algorithm

The metaclustering algorithm starts by creating numerous clusters from a data set. The dissimilarity between those clusters is then calculated, and a metacluster is generated as a result of that measurement. In this approach, the ensemble is represented by the final metacluster.

One of the most important characteristics of these algorithms is that the number of clusters that the algorithm will build must be declared at the start. For the specified data set, we used the cluster number created by the best distance-linkage combination.

### Observing Pleiotropy for Different Cancers

We identified the genes responsible for various cancer tumors from the data sets and then evaluated their expression in different patients with cancer to report their various phenotypes in order to discover the pleiotropic behavior of distinct genes. We built a secondary data set by extracting the expression data for each gene from each data set after identifying the common genes across these disorders. Every primary data set must contain an equal number of time stamp values in order to build a 2D microarray data set. The data sets, however, have different numbers of columns. Central nervous system, for example, includes 60 time stamps for a single gene, but the ALL-AML (acute lymphoblastic leukemia-acute myeloid leukemia) data set has 72 time stamps. We cannot modify or remove any columns from the data set because doing so could compromise the data’s integrity or result in the loss of valuable information. To address this issue, we estimated the mean, median, standard deviation, and variance, which may be used to summarize numerical data [30], and we used these numbers to construct our secondary data set. We will design a hierarchical tree using the perfect distance-linkage method found in the previously presented method because we have a data set for each gene with pleiotropic behavior. For that particular gene, the diseases that are closest to each other share similar summarized statistics. As a result, these trees will aid our understanding of how a single gene exhibits various phenotypes in patients with cancer. Furthermore, the gene enrichment scores of these common genes for the disorders that are frequent will be used to corroborate our findings.

### Ethical Considerations

Since no human or animal trial was conducted during this research, the authors did not apply for an ethical approval for the study.

## Results

We will discuss the experimental outcomes we discovered in our research in this part. We started by explaining the data sets we used. The findings for various distance-linkage method combinations were then shown. We later presented our findings in terms of pleiotropy for the shared genes.

### Data Set

We obtained gene expression data for various cancers from a publicly accessible database [31]. Every data set includes the disease-causing genes as well as their expression in various patients with the same condition. We also examined a data set from a variety of disorders to confirm that our findings were disease-agnostic. We used 7 data sets for various cancers. Table 2 lists the specifics of each data set.

The number of genes and patients, or the number of conditions for each gene, differs among these data sets. We used a diverse data set to discover the ideal metrics, which can be used to any gene expression data set. Furthermore, these databases contain certain genes that are widely used. We have created a secondary data set to explore and analyze those genes further.

**Table 2:** Description of data sets.

Data set	Data domain	Number of patients	Number of genes
CNS^a^	Central nervous system	60	7129
ALL-AML^b^	Acute lymphocytic leukemia	72	7129
Lung cancer	Lung cancer	181	12,533
Ovarian cancer	Ovarian cancer	253	15,154
Lymphoma	Lymphoma	62	4022
SRBCT^c^	Small round blue cell tumor	83	2308

^a^CNS: central nervous system.

^b^ALL-AML: acute lymphoblastic leukemia-acute myeloid leukemia.

^c^SRBCT: small round blue cell tumor.

### Result of Experiments for Identifying the Best Distance-Linkage Method

In our experiment, we employed several combinations of distance measurements and connection algorithms to generate a hierarchical tree. To validate our founding, we used 3 distance metrics and 4 linking methods. We combined these 3 distance metrics and 4 linkage methods to build 12 hierarchical trees for each data set. We cut each tree on numerous cut points after building hierarchical trees. As a result, the tree has been separated into several distinct groups. We assessed the fitness value for each cut point and selected the highest as the ideal value for that hierarchical tree given that particular distance metric-linkage method combination.

**Figure 2:**
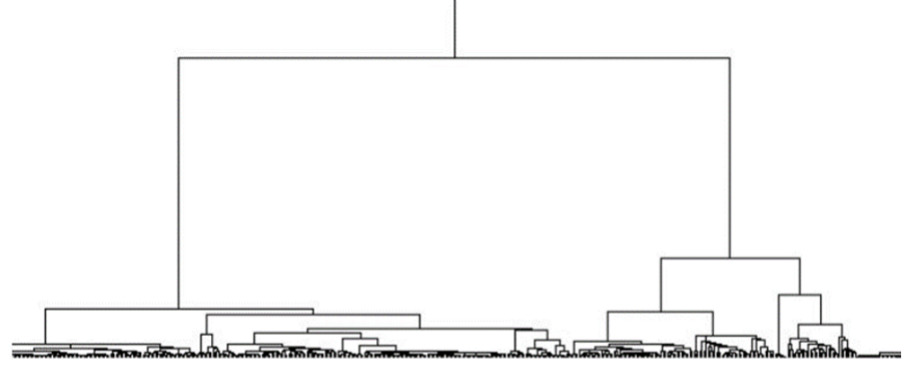
Hierarchical tree created using the maximum-Ward method on lung cancer data set.

A portion of a hierarchical tree of genes from the lung cancer data set is shown in Figure 2. This tree was constructed using the maximum-Ward combination. The full tree has a large number of leaves due to the data set’s 12,533 genes. All the values using Equation 13 are calculated, and the best values for each combination of distance method and linkage metric are shown in Table 3.

**Table 3:** Fitness value for different combinations of distance and linkage metrics.

Data set and linkage		Manhattan distance	Euclidean distance	Maximum distance
**CNS^a^**				
	Average	3.80×10^-13^	9.47×10^-12^	3.50×10^-11^
	Complete	1.42×10^-13^	6.78×10^-12^	3.44×10^-12^
	Single	2.59×10^-13^	5.72×10^-12^	2.16×10^-11^
	Ward	4.49×10^-14^	3.22×10^-13^	3.09×10^-12^
**ALL-AML^b^**				
	Average	1.20×10^-6^	1.45×10^-5^	3.39×10^-5^
	Complete	8.89×10^-7^	2.11×10^-5^	1.51×10^-5^
	Single	1.11×10^-6^	1.37×10^-5^	1.24×10^-5^
	Ward	4.41×10^-7^	2.64×10^-6^	3.07×10^-5^
**Lung cancer**				
	Average	5.56×10^-8^	1.48×10^-6^	3.36×10^-6^
	Complete	5.35×10^-8^	1.23×10^-6^	1.52×10^-9^
	Single	5.33×10^-8^	6.47×10^-7^	5.86×10^-7^
	Ward	3.03×10^-8^	1.19×10^-6^	6.71×10^-6^
**Ovarian**				
	Average	1.25×10^-5^	1.59×10^-4^	2.87×10^-4^
	Complete	1.71×10^-5^	7.49×10^-5^	6.28×10^-4^
	Single	2.88×10^-6^	3.12×10^-4^	1.28×10^-4^
	Ward	2.49×10^-4^	3.44×10^-5^	9.31×10^-4^
**Lymphoma**				
	Average	1.29×10^-7^	2.81×10^-6^	9.66×10^-6^
	Complete	2.21×10^-8^	2.34×10^-6^	6.00×10^-6^
	Single	1.01×10^-7^	2.81×10^-6^	8.10×10^-6^
	Ward	1.23×10^-8^	6.05×10^-7^	2.82×10^-6^
**SRBCT^c^**				
	Average	1.52×10^-7^	6.73×10^-6^	4.41×10^-5^
	Complete	1.03×10^-7^	4.72×10^-6^	3.73×10^-5^
	Single	8.24×10^-8^	4.34×10^-6^	3.00×10^-5^
	Ward	3.88×10^-9^	8.55×10^-8^	2.67×10^-6^

^a^CNS: Central Nervous System.

^b^ALL-AML: acute lymphoblastic leukemia-acute myeloid leukemia.

^c^SRBCT: small round blue cell tumor.

### Ensemble Result

We chose the data set (ALL-AML) for testing and ran these 4 ensemble clustering techniques. For this data set, the maximum-average combination produced the best result, with a cluster number of 135. Table 4 displays the fitness values. We discovered that no ensemble clustering approach improves fitness value in any way.

**Table 4:** Fitness value for different ensemble techniques.

Ensemble techniques	Fitness value
CSPA^a^	4.32×10^-6^
HGPA^b^	3.29×10^-6^
MCLA^c^	1.53×10^-5^
Maximum-average	3.39×10^-5^

^a^CSPA: cluster-based similarity partitioning.

^b^HGPA: hyper graph partitioning algorithm.

^c^MCLA: metaclustering algorithm.

### Result Analysis for Common Genes

**Figure 3:**
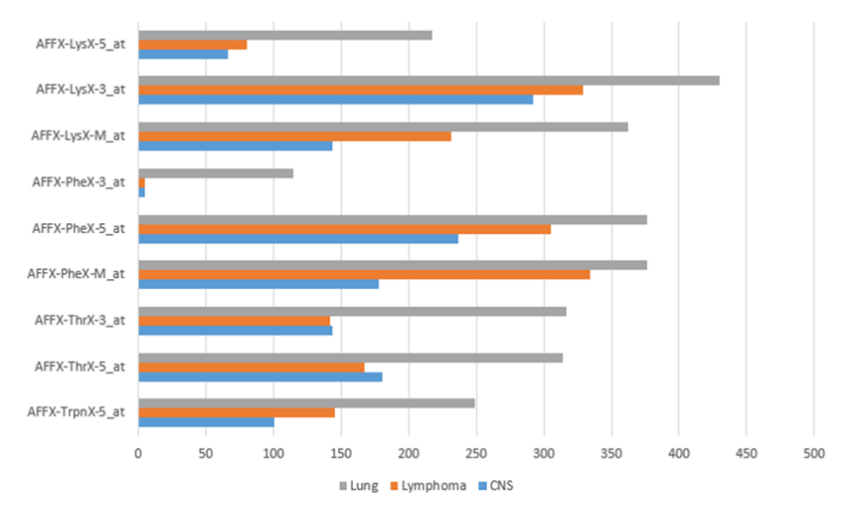
Gene enrichment score vs cancer type.

Multiple tumors can be caused by a small number of genes. We discovered 9 genes linked to the following 3 types of cancer: central nervous system, lymphoma, and lung cancer. AFFX-TrpnX-5 at, AFFX-ThrX-5 at, AFFX-ThrX-3 at, AFFX-PheX-M at, AFFX-PheX-5 at, AFFX-PheX-3 at, AFFX-LysX-M at, AFFX-LysX-3 at, and AFFX-LysX-5 at were discovered to be common genes. We found the gene enrichment score publicly available at [32] to confirm our findings. Gene enrichment scores in various malignancies are given in Figure 3 for the discovered common genes.

## Discussion

### Principal Findings

The maximum distance method combined with the average linkage method produces better hierarchical trees in 4 data sets (central nervous system, leukemia, lymphoma, and SRBCT), according to the fitness values provided in Table 3. These data sets are medium in size, with 60-80 rows and 2000-7000 columns, as shown in Table 2. In the Spellmen data set, however, the maximum-average combination also excels. The other 4 data sets reflect human genes that are responsible for specific tumors, whereas Spellmen is a microarray data set of bacteria. However, the maximum distance approach with ward linkage method constructs a superior hierarchical tree compared with the other methods in 2 of the largest data sets, lung and ovarian. These 2 data sets are larger than the others, and they share no genes with the others.

The maximum distance metric outperforms the other 2 distance methods among the 3 most commonly used distance metrics. Maximum distance considers only 1 column where those 2 genes have the most variance when calculating distance between them. The Euclidean and Manhattan distance methods, on the other hand, would have taken distances across all columns. As a result, the dissimilarity values for the Euclidean and Manhattan distances are approaching the maximum distance. As a result, in clustering, the Euclidean and Manhattan distances place points slightly farther apart than the Maximum distance. Furthermore, because all the columns indicate the same features of a gene evaluated at different time stamps, we can analyze the worst scenario (ie, the greatest differential in the expression of 2 genes at a certain moment). This is the most significant difference between these 2 genes. To put it another way, maximum distance calculates only the difference that matters. The Euclidean and Manhattan distances, on the other hand, are becoming buried in the massive amount of data. The maximum distance, on the other hand, may create undesirable clusters in a different data set with uniform variation across all columns.

When the data set is small, the average linkage approach performs well, and when the data set is huge, the ward method performs well. The single linkage approach may be faster than the average method for joining clusters, but it is not necessarily better. When determining the proximity of 2 clusters, it always considers only 2 points and ignores all others. The average linkage approach, on the other hand, considers all the points in the cluster when determining relatedness. When using the ward technique, the sum square error is used to determine similarity. When working with small or medium-sized data sets, the average linkage approach outperforms the ward linkage method, but as the data sets grow larger, the sum square error values take over and produce superior results compared with the average linkage method.

We tried to identify the optimal combination in our research and found that the maximum distance method performs better on hierarchical clustering when column variance is not uniform across the data set. However, if the data set is medium in size, with around 2000-7000 rows and 60-80 columns, the average linkage technique will outperform other linkage methods, and if the data set is very large, with 12,000-15,000 rows and 100-200 columns, the ward linkage approach will outperform other linkage methods. Furthermore, it has been discovered that ensemble clustering can improve performance by a very little amount at the cost of extra work.

We discovered 9 common genes that cause the following 3 diseases: lymphoma, central nervous system cancer, and lung cancer. We tried to figure out how these genes play a role in these 3 diseases using the data provided in the data sets. The maximum-average hierarchical clustering technique was chosen since it performed the best in the first experiment. We used gene enrichment score to confirm our findings on whether the 9 genes discovered have an impact on these 3 conditions. Figure 3 shows the gene enrichment scores for these genes. We can see that 8 of the 9 genes are important for all 3 cancers. Only 1 gene (AFFX-PheX-3 at) is more important than the other 2 in lung cancer. However, it is clear that our discovered genes have a significant impact on these 3 cancer forms.

Bioinformatics is becoming more and more involved in health sectors, such as disease detection and individualized medicine recommendation, as computational technology advances. Clustering techniques are becoming increasingly important in these industries. We investigated several distance-linkage combinations and attempted to find a solution. We hope that other researchers who use hierarchical clustering will profit from our findings and apply what they have learned to their own study. We also discovered common genes with multiple symptoms, which we confirmed using gene enrichment profiling. Knowing the pleiotropic nature of these genes will help scientists work on them to combat cancer.

### Conclusion

In this study, we discovered a set of measures that will yield higher-quality clusters for gene expression data. Pleiotropic behavior of common genes for many disorders was also discovered. To validate our findings, we used a variety of data sets that varied in size and richness. We used a fitness function to compare cluster quality between sets of clusters while assessing cluster quality. For medium-sized data sets, we discovered that the maximum distance metric combined with average linkage works best. Ward linkage also works better with huge data sets. Furthermore, due to data dimension differences, we had to preprocess data while identifying common genes for various disorders. It is critical to identify genes with similar symptoms more precisely and to separate those genes more effectively. Furthermore, detecting a gene by applying the clustering technique to find comparable genes is a critical work for researchers, and if done correctly, might save countless lives. For all these reasons, correct clustering is becoming increasingly important in bioinformatics. Therefore, if their data set resembles our microarray data, researchers from other fields can employ this technology.

## Abbreviations

ALL-AML: acute lymphoblastic leukemia-acute myeloid leukemia
ASW: average silhouette width
